# A novel bispecific molecule delivered by recombinant AAV2 suppresses ocular inflammation and choroidal neovascularization

**DOI:** 10.1111/jcmm.13086

**Published:** 2017-03-22

**Authors:** Yiming Li, Ping Zhu, Amrisha Verma, Tuhina Prasad, Hongxin Deng, Dechao Yu, Qiuhong Li

**Affiliations:** ^1^ State Key Laboratory of Biotherapy and Cancer Center West China Hospital Sichuan University, and Collaborative Innovation Center for Biotherapy Chengdu Sichuan China; ^2^ Department of Ophthalmology College of Medicine University of Florida Gainesville FL USA

**Keywords:** choroidal neovascularization, angiogenesis, age‐related macular degeneration, ocular inflammation, gene therapy, AAV, vascular endothelial growth factor, complement, uveitis, autoimmune uveitis

## Abstract

Elevated vascular endothelial growth factor (VEGF) and complement activation are implicated in the pathogenesis of different ocular diseases. The objective of this study was to investigate the hypothesis that dual inhibition of both VEGF and complement activation would confer better protection against ocular inflammation and neovascularization. In this study, we engineered a secreted chimeric VEGF inhibitor domain (VID), a complement inhibitor domain (CID) and a dual inhibitor (ACVP1). Vectors expressing these three inhibitors were constructed and packaged into AAV2 (sextY‐F) particles. The expression and secretion of the proteins were validated by Western blot. The effects of these inhibitors expressed from AAV2 vectors were examined in endotoxin‐induced uveitis (EIU), experimental autoimmune uveoretinitis (EAU) and choroidal neovascularization (CNV) mouse models. The AAV2 vectors expressing the CID‐ and ACVP1‐attenuated inflammation in EIU and EAU model, whereas the vector expressing VID showed improved retinal structure damaged by EAU, but not affect the infiltration of inflammatory cells in EAU or EIU eyes. Both VID and CID vectors improved laser‐induced retinal and choroid/RPE injuries and CNV, whereas ACVP1 vector provided significantly better protection. Our results suggest that gene therapy targeting VEGF and complement components could provide an innovative and long‐term strategy for ocular inflammatory and neovascular diseases.

## Introduction

Ocular inflammatory and neovascular diseases are the leading causes of blindness. Retinal and choroidal vascular diseases constitute the most causes of moderate and severe vision loss in patients with ocular disease 1.

Uveitis is a severe inflammatory eye disease which accounts for 5–10% of preventable blindness in United States, 10–15% of all cases of blindness worldwide, and affects individuals of all ages, genders and races 2, 3. Corticosteroids, macrolides and other immunomodulatory agents are mainly employed in the current treatment 4, 5, most of which are reported to cause severe complications such as cystoid macular oedema, choroidal neovascularization (CNV), retinal neovascularization (RNV) and global immunosuppression 6. The pathogenesis of uveitis could be complicated as it encompasses a wide range of underlying aetiologies. Several animal models, such as experimental autoimmune uveitis (EAU), endotoxin‐induced uveitis (EIU), have been used for understanding disease pathogenesis and testing new therapies 7. Data from clinical cases and animal models provide strong evidence indicating that anti‐inflammation could be main therapeutic target in the uveitis treatment 5 and vascular endothelial growth factor (VEGF) inhibition could be considered as an option for treating uveitis 8.

Age‐related macular degeneration (AMD) is the leading cause of vision loss in United States, Europe and Australia. AMD begins with the appearance of lipoproteinaceous deposits between the RPE and Bruch's membrane. Experimental and clinical study has revealed that immunological processes such as the recruitment of macrophages, complement activation and microglial activation are involved in early stage 9, 10, 11, 12. However, approximately 10% of patients can progress towards wet or exudative AMD, which causes 90% vision loss of AMD patients. Evidence from experimental model and clinical trials highlighted that retinal hypoxia, which leads to overproduction of cytokines such as VEGF, plays a critical role in both RNV and CNV, the ‘wet form’ of age‐related macular degeneration (AMD).

Vascular endothelial growth factor is a critical cytokine involved in angiogenesis. Physiologically, VEGF level is controlled by endogenous angiogenic inhibitors. However, this homeostasis could be disturbed in retinal and choroidal neovascular diseases 13, 14, 15. Although the underlying mechanism of CNV development is still unclear, it has been shown by previous studies that CNV could be arrested when VEGF is inhibited 16. Several biological agents targeting VEGF are approved by US Food and Drug administration (FDA) in the treatment of various ocular diseases 17. Furthermore, increasing reports show that VEGF plays an important role in the pathogenesis of uveitic complications 18. Although the direct anti‐inflammation effect of anti‐VEGF treatment has not been completely validated, it is expected to provide a prospective two pronged treatment regimen with immunosuppressive agent.

The complement cascade is known to play an important role in innate immunity. The inappropriate activation of complement cascade has been implicated in various ocular diseases 9. Previous studies revealed that the severity of uveitis was dramatically reduced when C3, the central component of complement cascade, was inhibited 19, 20. Furthermore, anaphylatoxins C3a and C5a were present in drusen of age‐related macular degeneration patients and induced VEGF expression *in vitro* and *in vivo* 21. In addition, membrane attack complex (MAC) accumulated in the process of CNV in the experimental animals 22. CNV is found to be significantly reduced when complement component is blocked or deleted 23, which is an indirect indication that the dysregulation of complement cascade could result in their aggregation in the local environment of CNV.

We hypothesize that dual inhibition of VEGF and complement component would provide a more potent anti‐angiogenesis and anti‐inflammation protection for ocular neovascular and inflammatory diseases. We designed a VEGF inhibitor domain (VID) by a decoy receptor linked to the IgG constant region (Fc). VID has the same sequence of the VEGF trap (aflibercept) which consists of the second immunoglobulin (Ig)‐like domain of vascular endothelial growth factor receptor 1 (VEGFR1) and the third immunoglobulin (Ig)‐like domain of VEGFR2. VEGF trap was shown to reduce CNV and improve visual acuity in preclinical animal models and approved in phase III clinical trials of patients with age‐related macular degeneration 24 and phase I clinical trial of patients with diabetic macular oedema 25. We also designed a complement inhibitor domain (CID) derived from soluble complement receptor 1 (sCR1). As a variant of sCR1, CID has high‐affinity binding with complement C3b and complement C4b, and could inhibit both classical and alternative complement pathways. We further designed a dual decoy receptor (ACVP1) by fusing VEGF binding motif at the N terminal of Fc and complement binding motif at C terminal of Fc. ACVP1 is able to inhibit both VEGF and complement pathway with similar affinity as that of single inhibitor. The engineered VID, CID and ACVP1 fusion genes were then cloned into adeno‐associated virus (AAV) viral vectors and packaged into AAV2. The anti‐inflammation and angiogenesis efficacy was tested in the rodent model of ocular inflammation and CNV. Viral delivery of ACVP1 conferred a protection against inflammation in the EIU and EAU model, and reduced the CNV formation in laser‐induced CNV model, which could serve as a potential strategy for uveitis, AMD and other ocular inflammatory and neovascular diseases.

## Materials and methods

### Vector construction

The VID was designed to contain the second immunoglobulin (Ig)‐like domain of VEGFR1 gene, the third immunoglobulin (Ig)‐like domain of VEGFR2 gene and IgG Fc domain gene. The CID was designed to contain the gene of IgG Fc domain linked with first three sCR1 domains with N29K, S37Y, G79D and D109N amino acid substitution mutation which enhance the binding activity to C3b and C4b. The dual inhibitor, ACVP1, is a chimeric gene with IgG Fc sequence linked between VEGFR‐like domain sequence and complement receptor‐like domain sequence. All the inhibitor genes are preceded by the IgG signal peptide for secretion. All the AAV vectors were constructed using the AAV plasmid under the control of the chicken β‐actin (CBA) promoter and packaged into serotype 2 viral particles. AAV viral particles were produced, purified and titrated by the Vector Core of the Center for Vision Research at the University of Florida. The plasmid containing green fluorescent protein (GFP) driven by CBA promoter was packaged as control vector.

### *In vitro* characterization of VID, CID and ACVP1 expressed from AAV

HEK 293 cells were plated in six‐well plates with Dulbecco's Modified Eagle Medium (Thermo Fisher Scientific, Waltham, MA, USA) containing 10% foetal bovine serum (Thermo Fisher Scientific, Waltham, MA, USA). The media were replaced with media containing AAV at a multiplicity of infection (MOI) of 6000 when the cells grew to 70% confluence. Supernatant was collected 72 hrs after AAV infection followed by 6200 × g centrifugation.

### Western blot

Tissues were analysed and homogenized in RIPA Buffer (Sigma‐Aldrich, St Louis, MO, USA), containing 1% protease inhibitor cocktail (Thermo Fisher Scientific, Rockford, IL). Samples were kept on ice for 30 min. followed by centrifugation at 12,000 × *g* for 15 min. at 4°C. The supernatant was tested for protein concentration by BCA Assay (Thermo Fisher Scientific, Rockford, IL, USA). Cells and supernatant samples, prepared as described above, were electrophoresed on SDS polyacrylamide gel and transferred onto PVDF membrane. The membrane was probed with primary antibody and a secondary antibody conjugated to IR‐Dye (Li‐Cor Bioscience, Lincoln, NE, USA). The results were detected by Odyssey imaging scanner (Li‐Cor).

### Animals and experimental procedures

All animal experiments were conducted according to ARVO statement for the use of animals in Ophthalmic and Vision Research, and the protocol was approved by the Animal Care and Use Committee of University of Florida. The animals were kept with standard laboratory chow and water in air‐conditioned room with a 12‐hrs light–12‐hrs dark cycle. Mice were randomly divided into six groups: control (without any treatment), uninjected, AAV‐control (expressing GFP), AAV‐VID, AAV‐CID and AAV‐ACVP1. All the vectors were injected intravitreally at a dose of 7.5 × 10^8^ vg per eye.

### EIU mouse model

Six to seven‐week‐old C57BL/6J mice were used in this experiment. Mice were anaesthetized with ketamine and xylazine, and given an intravitreal injection of different vectors at the dose of 7.5 × 10^8^ vg in each eye. Ocular inflammation was induced by a single intravitreal administration of 25 ng lipopolysaccharide (LPS; Sigma‐Aldrich) 3 weeks after AAV vector injections. Mice were killed 24 hrs after LPS administration when the inflammation peaked 26, and eyes were carefully enucleated and processed for evaluation.

### EAU mouse model

Eight to 10‐week‐old B10.RIII mice were randomly divided into different groups and received viral treatments as described above. EAU was induced by immunization with 50 μg of IRBP (161–180) (SGIPYIISYLHPGNTILHVD) (Genscript, Piscataway, NJ, USA) with CFA (Sigma‐Aldrich) (1:1 vol/vol) subcutaneously 27 3 weeks after viral administration. Fundus and OCT were performed non‐invasively to monitor the inflammation 14 days after the immunization. Mice were killed, and eyes were enucleated for histopathology at day 15 of EAU induction.

### Laser‐induced choroidal neovascularization mouse model

Adult C57BL/J (8–10‐week‐old) mice were used in this experiment and were given intravitreal AAV injections as described above. CNV was induced by laser injury of Bruch's membrane 3 weeks after AAV injection using a 532‐nm‐wavelength diode laser with power parameters of 50 μm spot size, 0.1 sec. exposure and 200 mW. Laser photocoagulation was delivered through a slit lamp with a cover slide as a contact lens. A pattern of five lesions was placed concentrically around the optic nerve. Only the burns that produced a bubble confirming the success of laser induction were included in the study 28. Fundoscopy, fluorescence angiography and SD‐OCT were used to assess the development of CNV 10 days after laser treatment. The quantitative evaluation of laser‐induced CNV was performed 14 days after laser. Mice were deeply anaesthetized and perfused by 5 mg/ml 2000KD FITC conjugated dextran (Sigma‐Aldrich). Eyes were enucleated and fixed in 4% paraformaldehyde for 1 hr. Choroid and retinal flat mounts were prepared by carefully removing the excess periocular muscles as described previously 29.

### Fundoscopy and SD‐OCT

Fundus imaging with a Micro III retinal imaging microscope (Phoenix Research Laboratories, Pleasanton, CA, USA) was used to assess retinal pathology in EAU and laser‐induced CNV model. The pupils were dilated by 1% atropine and 2.5% phenylephrine. Mice were anaesthetized with a mixture of ketamine (75 mg/kg) and xylazine (5 mg/kg) in PBS intraperitoneally, and 2.5% hypromellose demulcent ophthalmic solution (Akorn, Buffalo Grove, IL, USA) was used to keep moist on ocular surface.

In the laser‐induced CNV model, fluorescence angiography was performed, when mice were injected with 150 Kd FITC‐conjugated dextran (25 mg/kg) via infraorbital plexus veins, and fundus images were taken as described above using the fluorescence filters.

Mice were dilated and anaesthetized as described above. To avoid loss of moisture from the ocular surface during procedure, mice received a drop of artificial tears (Systane Ultra, Alcon, Fort Worth, TX, USA). Spectral domain optical coherence tomography (SD‐OCT) images were captured by the Bioptigen Spectral Domain Ophthalmic Imaging System (Bioptigen, Durham, NC, USA). Images acquired by default software of the company.

In the EAU model, the average single B‐scan and volume scan were obtained when images centred on optical nerve head. The retinal thickness was measured from five frames of the volume of OCT images and averaged from the intensity peak of boundary corresponding to the vitreo‐retinal interface to the intensity peak corresponding to the retinal pigmented epithelium 30. In the laser‐induced CNV model, the average B‐scan and volume scan were obtained when images were focused on laser lesion.

### Histopathology evaluation

All the eyes used in the immunofluorescence or immunohistochemistry study were fixed in 4% paraformaldehyde freshly made in PBS overnight at 4°C and washed with PBS. Eyes were then cryoprotected in 30% sucrose and embedded into optimum cutting temperature compound (Tissue‐Tek; Sakura‐Finetek, Torrance, CA, USA). Eyes were sectioned at a thickness of 12 μm through the cornea‐optic nerve axis and mounted on SuperFrost Plus slides (Thermo Fisher Scientific). The slides were preheated at 37°C for 15 min. and permeated in 1% Triton X‐100 in PBS for 15 min. The slides were blocked with 5% BSA or 10% goat serum for 30 min. at room temperature. After that, tissue samples were incubated with different primary antibodies—rabbit anti‐VEGF (1:200; Santa Cruz Biotechnology, Santa Cruz, CA, USA), rabbit anti‐C5b‐9 (1:100, Abcam, Cambridge, MA, USA), rat anti‐CD31 (1:50; BD PharMingen, San Diego, CA, USA), rabbit anti‐cleaved Caspase‐3 (1:200; Millipore, Billerica, MA, USA), rabbit anti‐Iba1 (1:400, Wako chemicals, Richmond, VA) and FITC‐conjugated anti‐CD45 and anti‐CD11b (1:200, BD PharMingen, San Diego, CA, USA) at 4°C overnight and then incubated with appropriate secondary antibody conjugated to Alexa 488 or 594 (Molecular Probes/Invitrogen, Grand Island, NY, USA) or alkaline phosphatase (Sigma‐Aldrich) at 37°C for 1 hr. Sections for immunofluorescence were washed with 300 nM DAPI for 10 min. and mounted by Dako mounting media (Dako North America, Carpinteria, CA, USA) and finally imaged on a spinning disc confocal (UltraVIEW Vox, PerkinElmer, Waltham, MA, USA). These experiments are repeated by three sections from at least two mice in each group. Sections for immunohistochemistry were equilibrated with 0.1 M PH 9.5 Tris‐HCl buffer after secondary antibody incubation. NBT/BCIP was used to develop colour with levamisole as suppressor of endogenous phosphatase activity. The reaction was terminated when positive signal was observed in microscope. The slides were mounted by 50% glycerol and imaged by a Zeiss microscope (AxioVision, Carl Zeiss MicroImaging, Inc., Thornwood, NY, USA). Eyes were then either processed or embedded in paraffin. Sections were cut at a thickness of 5 μm and stained with haematoxylin and eosin (H&E). Images were captured by Zeiss microscope (AxioVision, Carl Zeiss MicroImaging, Inc., Thornwood, NY, USA). The infiltrated inflammatory cells in the anterior and vitreous chamber of the sections were counted under a light microscope.

### Statistical analysis

Data are expressed as the mean ± SD of at least two independent experiments. Differences between mean values of multiple groups were analysed by one‐way analysis of variance with Dunnett's test for *post hoc* comparisons. Statistical significance was reported whenever the calculated *P* value was ≤0.05. *P value ≤ 0.05, **P value ≤ 0.01, ***P value ≤ 0.001.

## Results

### Characterization of the transgene expression from recombinant Adeno‐Associated Virus *in vitro*

**Figure 1:**
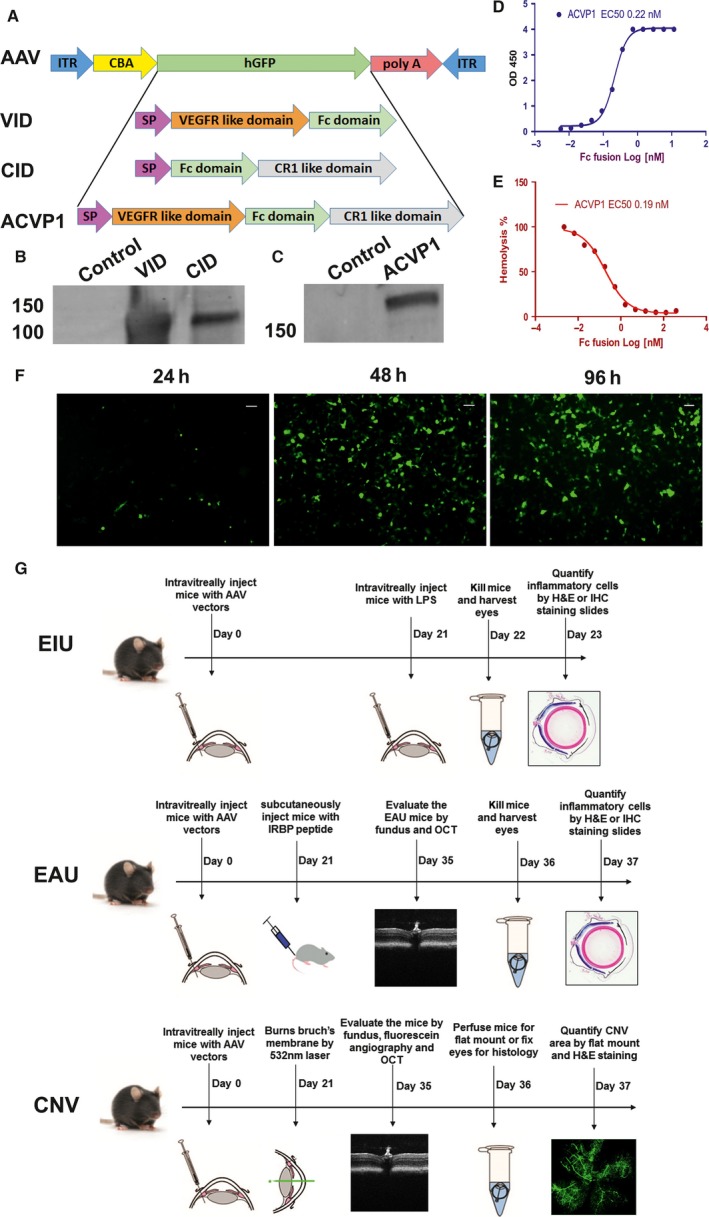
Construction and validation of AAV vectors expressing VEGF inhibitor domain (VID), complement inhibitor domain (CID) and ACVP1 genes. (**A**) Map of AAV constructs expressing VID, CID and ACVP1. The inhibitor sequences including the signal peptide sequence (SP) were cloned in an AAV plasmid between the chicken β‐actin (CBA) promoter and the SV40 poly A sequence of AAV. (**B**,** C**) HEK293 cells were infected with AAV vectors expressing VID, CID and ACVP1 genes. The supernatants from these cells were harvested, and the expression of different inhibitors was evaluated by Western blot. AAV vector expressing green fluorescent protein (GFP) was used as control vector to infect HEK293 cells. (**D**) The binding affinity of the chimeric dual inhibitor to VEGF165 was determined. ACVP1 (0–10 nM) were incubated with immobilized VEGF165 (30 nM), and binding affinity was determined by optical density at 450 nm (OD450). (**E**) The complement inhibition efficiency of chimeric dual inhibitor was determined by haemolysis assay. ACVP1 (0–500 nM) was incubated with normal human serum and then mixed with antibody‐sensitized sheep erythrocytes. The ratio of cell lysis was determined by optical density at 405 nm (OD405). (**F**) Efficiency evaluation of AAV2 (SextY‐F) infection on HEK 293 cells. Representative images taken at 24, 48 and 72 hrs after AAV2 (SextY‐F)‐GFP infection. Original magnifications 10X. Bar = 100 μm. (**G**) Experimental design for evaluation of the AAV2 (SextY‐F) vectors in the endotoxin‐induced uveitis, experimental autoimmune uveoretinitis and laser‐induced choroid neovascularization mouse model. Mice were injected intravitreally with 7.5 × 10^8^ vector genomes (vg) of AAV2 delivering VID, CID or ACVP1 three weeks before initiation in each model.

The VID was designed as a soluble decoy receptor, which consists of the second immunoglobulin (Ig)‐like domain of VEGFR1 and the third immunoglobulin‐like domain of VEGFR2 (Fig. 1A). VID has the same amino acid sequence of aflibercept (VEGF trap) with similar binding affinity (data not shown).

The CID was designed by fusing the soluble complement receptor 1 with IgG Fc. Dual inhibitor to both VEGF and complement (ACVP1) was constructed by fusing VEGF binding motif at N terminal and complement binding motif at C terminal (Fig. 1A). To suppress the level of VEGF and complement cascade in the retinal–choroidal tissue, the AAV vectors were designed to express these inhibitors from a ubiquitous promoter, the CMV enhancer and CBA promoter (Fig. 1A). The plasmids were packaged into capsid‐modified mutant AAV2 (Y252, 272, 444, 500, 704, 730F). This AAV2 (SextY‐F) variant can transduce different types of retinal cells more efficiently than wild‐type AAV2 as shown in previous studies 31, 32. The expression and secretion of the inhibitors were confirmed in cultured HEK293 cells 72 hrs after AAV vector infection (Fig. 1F). Supernatants were harvested, and proteins were analysed by Western blot using a goat anti‐human IgG Fc fragment antibody (Fig. 1B,C). The activity of the recombinant dual inhibitor molecule was further confirmed by ELISA and haemolysis assay, which showed that this molecular has an expected high affinity when binding to VEGF (Fig. 1D) and sufficient efficiency in suppressing haemolysis induced by complement cascade (Fig. 1E).

### Intravitreal administration of AAV‐CID and AAV‐ACVP1 vectors reduced endotoxin‐induced ocular inflammation

**Figure 2:**
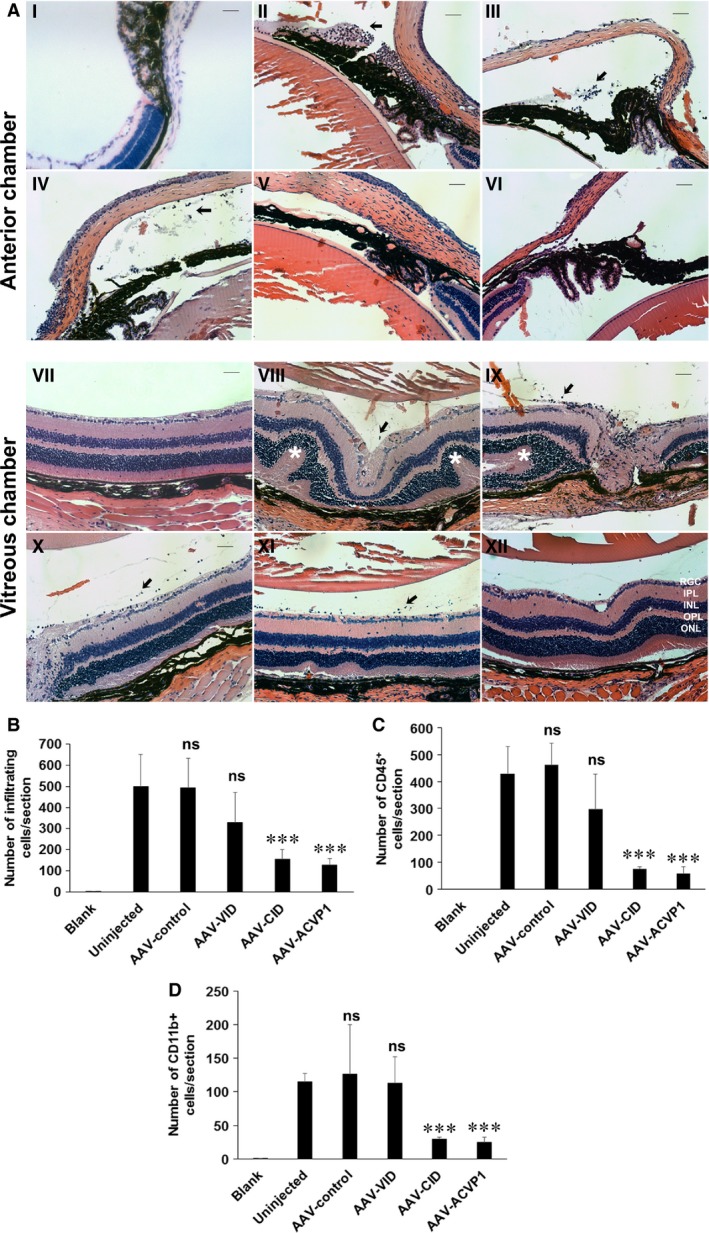
Histological and immunohistochemical evaluation of endotoxin‐induced uveitis (EIU) mice treated with VEGF inhibitor domain (VID), complement inhibitor domain (CID) and ACVP1 vectors. Mice were treated with control, VID, CID and ACVP1 viral vectors by intravitreal injection. Twenty‐one days after injection, LPS (25 ng/eye) was injected intravitreally to induce inflammation. Mice without AAV and LPS treatment were also included as control. (**A**) Representative H&E photographs of the anterior chamber (I to VI) and vitreous chamber (VI to XII) from control group (I, VII), untreated group (II, VIII), AAV‐control vector‐injected group (III, IX), AAV‐VID‐injected group (IV, X), AAV‐CID‐injected group (V, XI) and AAV‐ACVP1‐injected group (VI, XII). ONL, outer nuclear layer; OPL, outer plexiform layer; INL, inner nuclear layer; IPL, inner plexiform layer; RGC, retinal ganglion cell layer. Original magnifications 20×. Bar = 50 μm. Infiltrated inflammatory cells are indicated by arrow, and uveitis lesions are marked by star. Quantification of infiltrating inflammatory cells (**B**), CD45‐positive (**C**) and CD11b‐positive (**D**) cells. Values on *y*‐axis represent the number of infiltrating inflammatory cells/section. Results are given as mean ± SD; (*n* = 5 per group); ns means not significant *versus* uninjected group. *P < 0.05 (*versus* uninjected group). ***P < 0.001 (*versus* uninjected group).

The *in vivo* anti‐inflammatory effects of these AAV vectors were evaluated in a mouse model of EIU model. C57BL6J mice were randomly divided into following groups: (i) control, (ii) uninjected, (iii) control AAV vector (expressing GFP), (iv) AAV‐VID, (v) AAV‐CID, (vi) AAV‐ACVP1 group (*n* = 5 per group). AAV vectors were intravitreally injected at a dose of 7.5 × 10^8^ vector genome (vg) per eye. Similar vector dose expressing GFP resulted in efficient transduction of inner retina (Fig. S1). EIU was induced three weeks after AAV vector administration by intravitreal injection of lipopolysaccharide (LPS) at a dose of 25 ng per eye (Fig. 1G) as described previously 26. Severe uveitis was verified when massive leucocyte and monocyte infiltration was found in the iris, ciliary body, anterior chamber and vitreous chamber of the eyes 24 hrs after LPS injection as described previously 33. Sagittal sections of the eyes were stained by H&E, and infiltrated inflammatory cells were examined by bright‐field microscope. There was no significant difference in the number of infiltrating inflammatory cells in anterior chamber or vitreous chamber (Fig. 2A) between the uninjected and AAV‐control vector‐treated groups. AAV‐CID and AAV‐ACVP1 viral vectors, however, significantly reduced the number of infiltrating inflammatory cells in both anterior chamber and vitreous chamber, whereas AAV‐VID viral vector did not show any significant reduction in the number of inflammatory cells in anterior chamber or vitreous chamber as compared to control groups (Fig. 2B). CD45 and CD11b immunostaining was performed to analyse the different types of infiltrated cells. CD45 is known as leucocyte common antigen and is commonly used to identify inflammatory cells. CD11b is a marker of innate immune cells, that is monocytes, granulocytes and macrophages. CD45^+^ or CD11b^+^ cells are not found in the healthy vitreous cavity or anterior segment, but highly increased when serious inflammation exists. In comparison with the uninjected or AAV‐control group, AAV‐CID and AAV‐ACVP1 vector‐treated eyes showed significantly reduced CD45^+^ (Fig. 2C) and CD11b^+^ (Fig. 2D) cells. However, AAV‐VID‐treated eyes were not significantly different from untreated or control vector treated eyes in the number of infiltrating inflammatory cells.

### Intravitreal administration of AAV‐CID, AAV‐VID and AAV‐ACVP1 vectors attenuated experimental autoimmune uveitis

**Figure 3:**
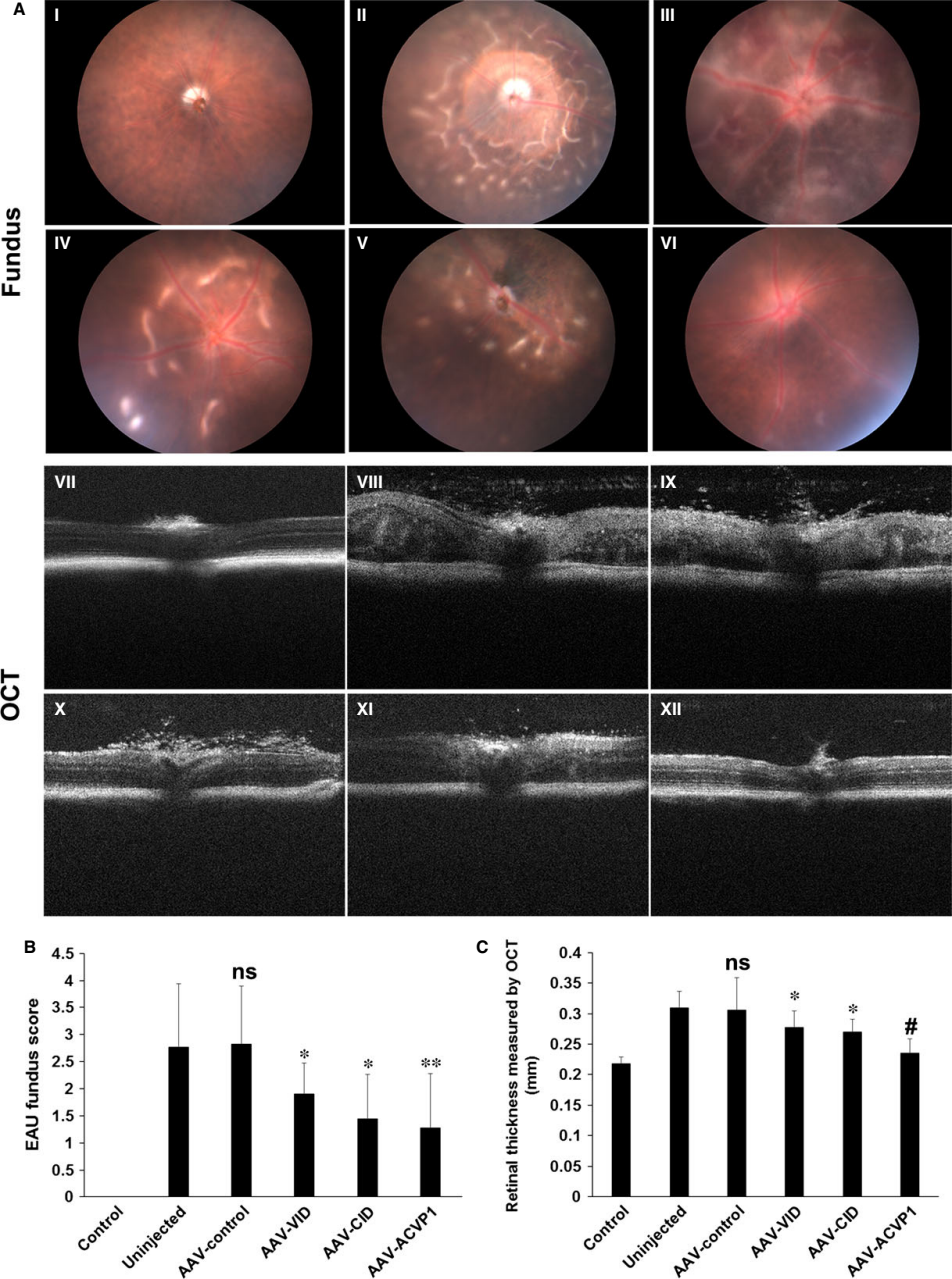
Clinical evaluation of experimental autoimmune uveoretinitis (EAU) from fundoscopic photographs and optical coherence tomography. Twenty‐one days after AAV treatment, mice were immunized by IRBP (161‐180) peptide. B10.RIII mice without AAV and IRBP (161‐180) peptide treatment were also included as control group. (**A**) Representative fundus (I to VI) and OCT (VII to XII) images of un‐immunized B10.RIII mouse eye (I, VII), untreated EAU (II, VIII) and AAV‐control vector‐treated (III, IX), AAV‐VEGF inhibitor domain (VID)‐treated (IV, X), AAV‐complement inhibitor domain (CID)‐treated (V, XI) and AAV‐ACVP1‐treated (VI, XII) EAU eyes. The extent of inflammatory reactions such as mild‐to‐severe vasculitis, focal lesion, large confluent lesions, retinal haemorrhages and folding, corneal oedema, etc., were observed in uninjected B10RIII mice by fundus and OCT images at the day 14 of EAU induction, all of which are significantly improved in vector‐treated eyes. (**B**) Clinical EAU score was evaluated on a scale of 0–4 based on fundus images. Values on *y*‐axis represent the average of clinical scores. Results are given as mean ± SD (*n* = 5 per group); ns means not significant *versus* uninjected group. *P < 0.05 (*versus* uninjected group). **P < 0.01 (*versus* uninjected group) (**C**) Retinal thickness measured from OCT images. Values on *y*‐axis represent the average of retinal thickness calculated manually from B‐scan OCT images. Results are given as mean ± SD (*n* = 5 per group); ns means not significant *versus* uninjected group. *P < 0.05 (*versus* uninjected group). #*P* < 0.05 (*versus* AAV‐VID and AAV‐CID groups).

Autoimmune susceptible B10.RIII mice were used for this study. The mice were divided into five groups and received the same doses of intravitreal injection of AAV vectors as in EIU model. Three weeks after viral administration, the peptide derived from human interphotoreceptor retinoid‐binding protein (IRBP161‐180) emulsified in complete Freund's adjuvant (CFA) was injected subcutaneously to induce EAU (Fig. 1G) 26, 27. Mice were examined by fundoscopy and spectral domain optical coherence tomography (SD‐OCT) on 14 days after IRBP161‐180 peptide injection, when the ocular inflammation reached peak levels. Funduscopic examination revealed evident inflammatory reactions such as mild‐to‐severe vasculitis, vitritis, large confluent lesions, retinal haemorrhages and folding, corneal oedema, etc., in the untreated group and control vector‐treated group (Fig. 3A). Clinical scoring using the criteria reported previously 34 were also applied to evaluate the therapeutic effect of vectors (Fig. 3B). Intravitreal cellular infiltration, retinal vasculitis, disorganized retinal layers, retinal folds and oedema in the retina can also be visualized by OCT imaging. Retinas in the EAU mice showed increased thickness due to retinal folds and oedema as measured by OCT imaging (Fig. 3A,C). Eyes that received intravitreal administration of AAV‐VID or AAV‐CID had significant decrease in retinal incrassation. More importantly, AAV‐ACVP1 conferred a better improvement evaluated by OCT images compared to the AAV vector expressing either CID or VID alone. These results indicated that VEGF‐A and Complement 3b/4b were both implicated in the pathogenesis of EAU.

**Figure 4:**
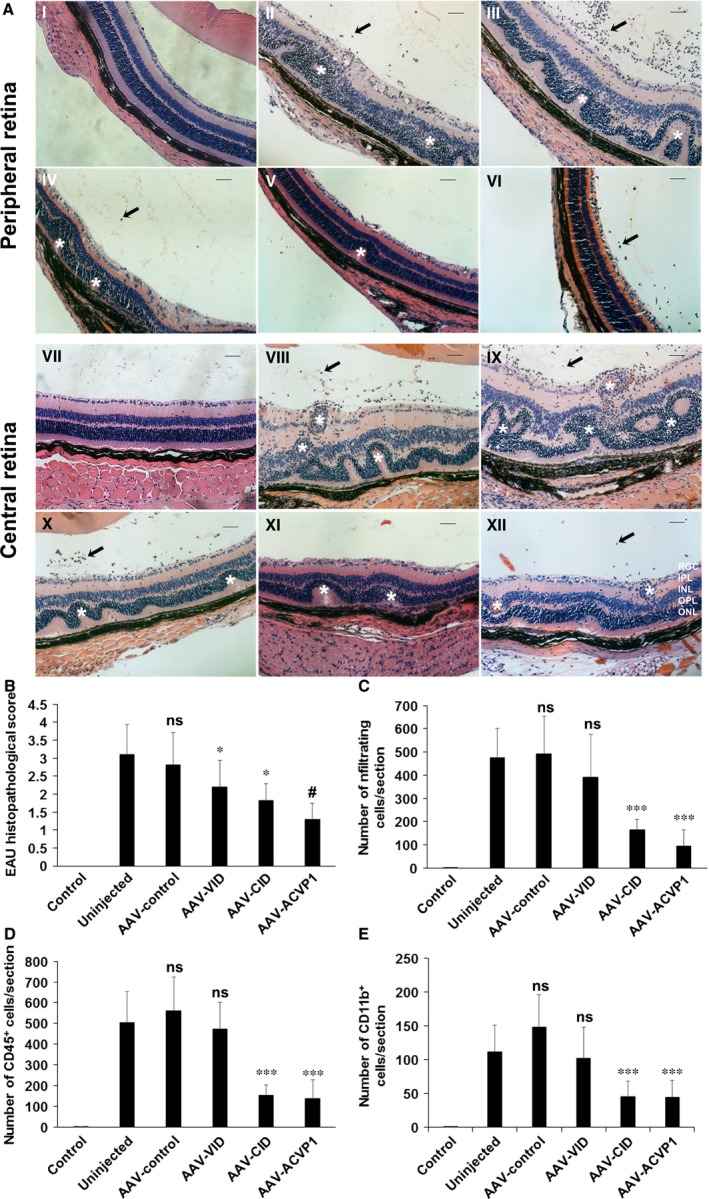
Histological and immunohistochemical evaluation of experimental autoimmune uveoretinitis (EAU) mice treated with VEGF inhibitor domain (VID), complement inhibitor domain (CID) and ACVP1 vectors. (**A**) Representative H&E staining images of peripheral (I to VI) and central retina (VII to XII) from unimmunized B10.RIII mouse eye (I, VII), untreated EAU (II, VIII), AAV‐control vector‐treated (III, IX), AAV‐VID‐treated (IV, X), AAV‐CID‐treated (V, XI) and AAV‐ACVP1‐treated (VI, XII) EAU eyes. ONL, outer nuclear layer; OPL, outer plexiform layer; INL, inner nuclear layer; IPL, inner plexiform layer; RGC, retinal ganglion cell layer. Magnification 20× Bar, 50 μm. Infiltrated inflammatory cells are indicated by arrow, and uveitis lesions are marked by star. (**B**) Histological EAU score was evaluated on a scale of 0–4. Values on *y*‐axis represent the average of clinical scores given on H&E stained images. Results are given as mean ± SD (*n* = 5 per group); *P < 0.05 (*versus* uninjected group). #*P* < 0.05 (*versus* AAV‐VID and AAV‐CID groups). Quantification of infiltrated inflammatory cells (**C**), CD45‐positive (**D**) and CD11b‐positive (**E**) cells. Values on *y*‐axis represent the number of infiltrating inflammatory cells/section. Results are given as mean ± SD (*n* = 5 per group); *P < 0.05 (*versus* uninjected group) ***P < 0.001 (*versus* uninjected group); ns means not significant.

Histopathological evaluation was performed on ocular sections. Massive infiltration of inflammatory cells, intensive retinal vasculitis and changes in the retinal thickness, folding of retina, as well as photoreceptor damage, were observed on the H&E‐stained sections in both uninjected group and AAV‐control group. Amelioration in retinal disorganization and inflammation was observed in all three vector (AAV‐VID, AAV‐CID and AAV‐ACVP1)‐treated groups (Fig. 4A). Quantitative histopathological grading (Fig. 4B) using the criteria reported previously 34 showed that both AAV‐CID vector and AAV‐VID vector had significant improvement in histopathological scores, and AAV‐ACVP1 provided even better protection from inflammatory damage than vector expressing VID or CID alone. Both AAV‐CID‐ and AAV‐ACVP1‐treated eyes had a dramatic decrease in the number of infiltrated inflammatory cells counted by H&E staining (Fig. 4C). However, no significant difference was found in the number of infiltrated inflammatory cells between AAV‐VID‐treated group and untreated group or the control vector‐treated group, and similar results were observed from CD45^+^ (Fig. 4D) and CD11b^+^ cell (Fig. 4E) analysis.

### Evaluation of AAV‐CID, AAV‐VID and AAV‐ACVP1 vectors in the laser‐induced CNV model

**Figure 5:**
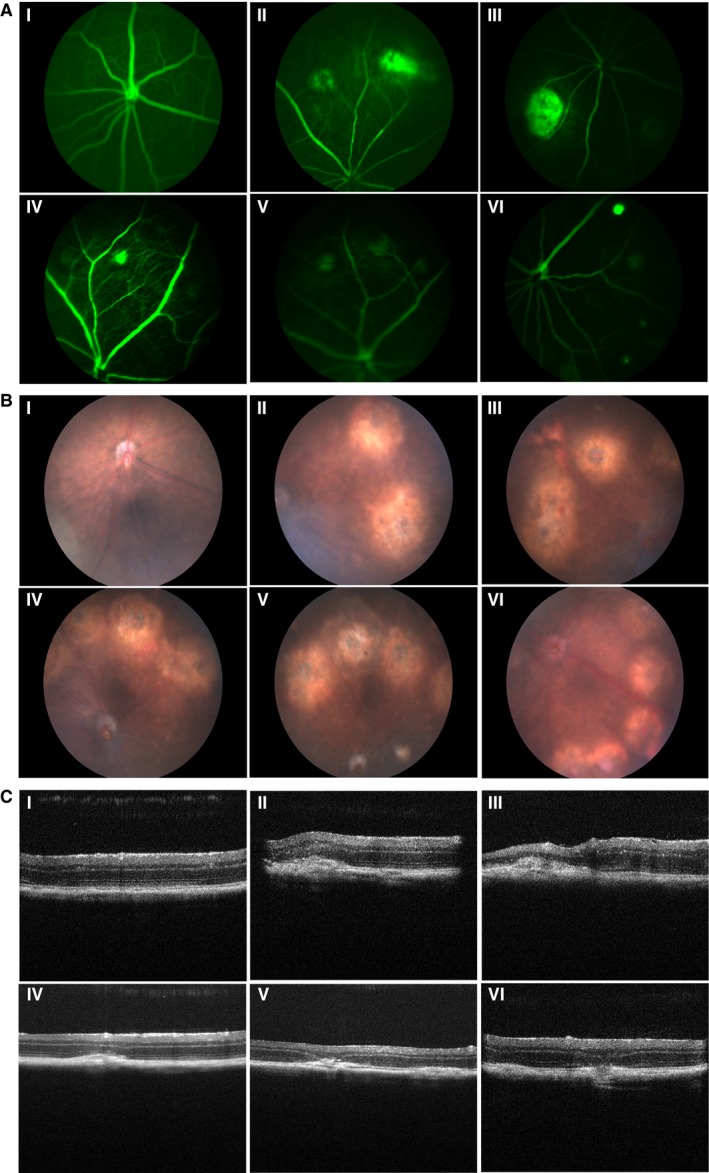
Non‐invasive evaluation of laser‐induced choroidal neovascularization (CNV) by fluorescence angiography, fundoscopy and optical coherence tomography. Mice were treated with 532 nm laser 21 days after AAV injection. C57BL/6J mice without any AAV and laser treatment were also included as control group. Fundus (I to VI), fluorescence angiography images (VII to XII) and cross‐sectional OCT images (XIII to XVIII) were obtained on day 10 after laser injury. Representative (**A**) fluorescence angiography, (**B**) fundus and (**C**) B‐scan images from control group mice (I, VII, XIII), uninjected mice (II, VIII, XIV), control AAV‐treated (III, IX, XV), AAV‐ VEGF inhibitor domain (VID)‐treated (IV, X, XVI), AAV‐complement inhibitor domain (CID)‐treated (V, XI, XVII) and AAV‐ACVP1‐treated (VI, XII, XVIII) mice.

**Figure 6:**
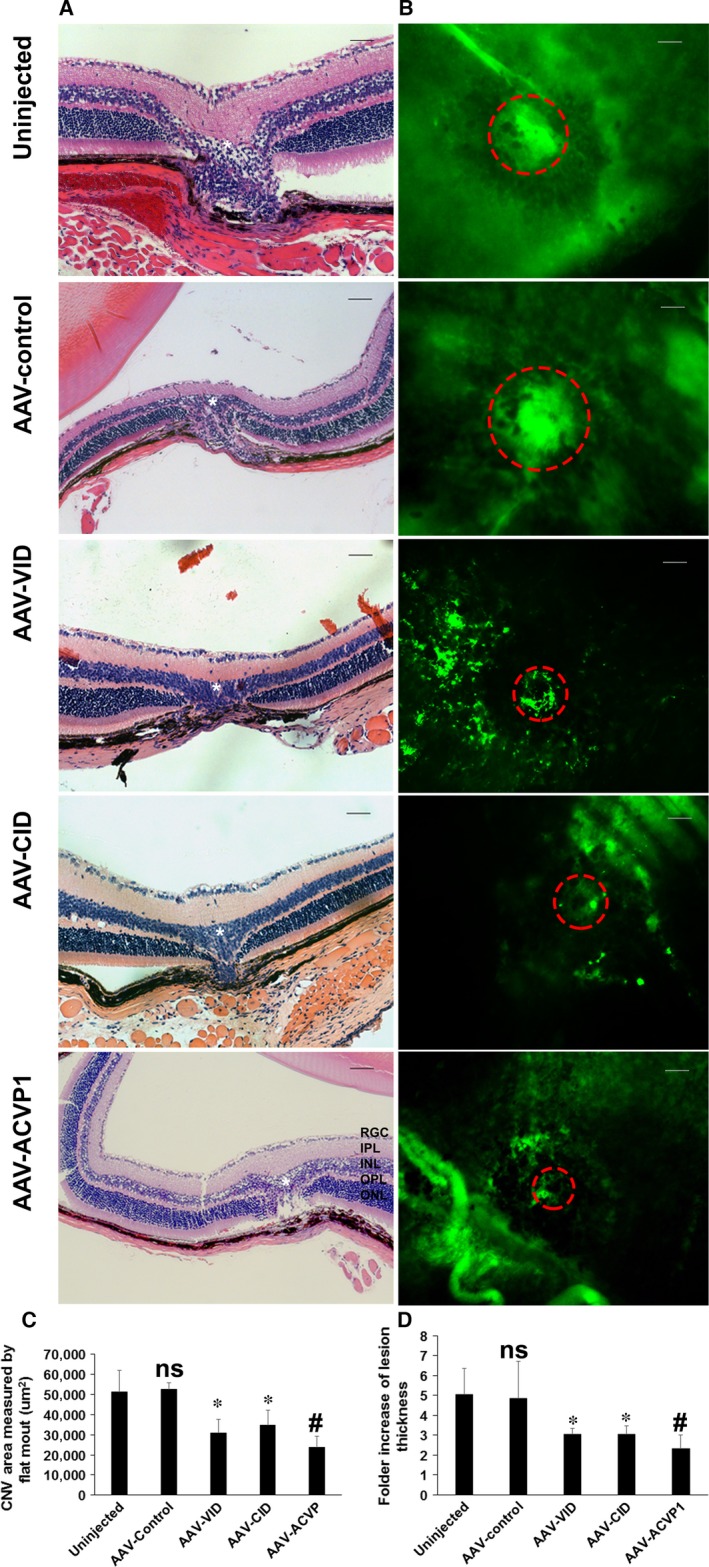
Assessment of choroidal neovascularization (CNV) area by H&E staining and choroid/RPE flatmount of laser‐induced CNV mice. (**A**) Representative images of H&E staining taken by Zeiss microscope. ONL, outer nuclear layer; OPL, outer plexiform layer; INL, inner nuclear layer; IPL, inner plexiform layer; RGC, retinal ganglion cell layer. Original magnification 20× Bar = 50 μm. The laser lesions were marked by star, and the choroid neovascularization were indicated by dashed circles. (**B**) Representative choroid/RPE flatmount images were taken by Zeiss fluorescence microscope, and (**C**) area were measured by Zeiss AxioVision Software measurement tool. Original magnification 20× Bar = 100 μm. Values on *y*‐axis represent CNV area (μm^2^). (**D**) Statistical analysis of neovascular lesion thickness from each group. Ratio of lesion thickness to adjacent normal thickness is measured. Values on *y*‐axis represent fold increase in lesion thickness. Data expressed as mean ± SD; (*n* = 5 per group); ns means not significant *versus* uninjected group *P < 0.05 (*versus* uninjected group). #*P* < 0.05 (*versus* AAV‐VID and AAV‐CID group).

C57BL6J mice were divided into five groups as described above, and each group included eight mice. Mice received similar viral treatment as in uveitis models. Three weeks after AAV administration, CNV was induced by 532‐nm diode laser, and the laser injuries were recorded immediately by funduscopic examination (Fig. S2a). Ten days postlaser injury, when neovascularization was well developed in mouse model as previous study had shown 28, bright‐field fundus photography and fluorescence angiography images were taken (Fig. 1G). Eyes treated with AAV‐VID and AAV‐CID vectors had a significant reduced area of neovascularization and decreased leakage compared to untreated eyes or control vector‐treated eyes (Fig. 5A and Fig. S2b, S2c). However, vector expressing dual inhibitor (AAV‐ACVP1) had apparently even better protection than the two mono‐inhibitor vectors (Fig. 5A,B and Fig. S2b). The extent of angiographically measured lesions was also confirmed by OCT examination and histological evaluation. Neuroretina oedema, abnormal hyperblastosis and loss of outer nuclear layer due to CNV were observed in the untreated group and control vector‐treated group by OCT images 10 days after laser injury (Fig. 5C) and H&E staining images 14 days after laser injury (Fig. 6A). However, groups that received vectors expressing different inhibitors all showed an improvement in the retinal and choroidal structure. More importantly, AAV‐ACVP1 vector had better therapeutic effects than mono‐inhibitor vectors. In the H&E staining images, the typical lesion and vascular lesion thickness were significantly reduced by viral vector treatment (Fig 6A, Fig S2d). In AAV‐ACVP1‐treated eyes, distinct retinal and choroidal structures could be recognized and there is no obvious hyperblastosis (Fig. 6A,D and Fig. S2d). CNV lesions were also assessed by FITC–dextran‐perfused RPE–choroid flat mounts. Significant CNV reduction was observed in eyes intravitreally injected with AAV‐VID or AAV‐CID compared to eyes without injection or with AAV‐control injected which is consistent with previous finding 35, 36. More interestingly, eyes that received an intravitreal injection with AAV‐ACVP1 had a much more reduction of CNV area comparing to eyes injected with AAV‐VID or AAV‐CID (Fig. 6B,C).

**Figure 7:**
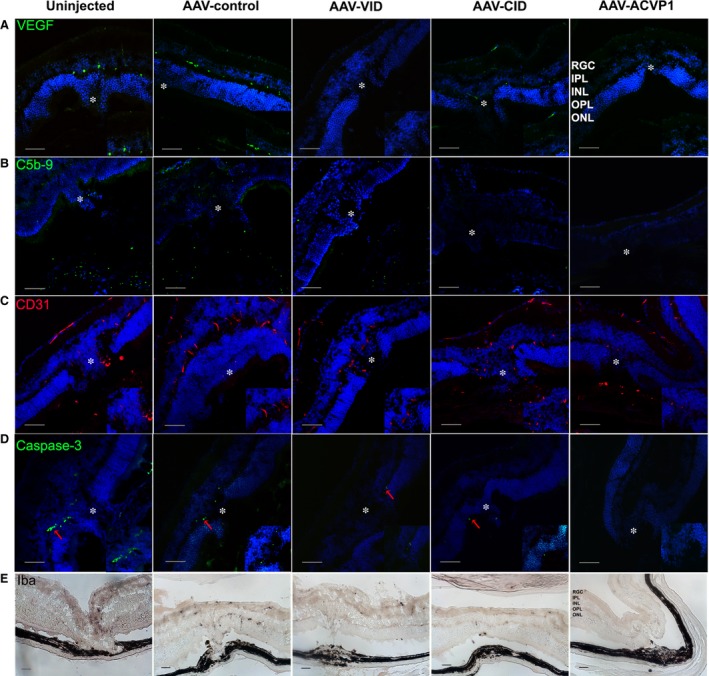
Immunofluorescence and immunocytochemistry evaluation of laser‐induced choroidal neovascularization (CNV) in untreated and inhibitor‐treated eyes. Immunofluorescence detection of (**A**) vascular endothelial growth factor (VEGF)‐a (green), (**B**) C5b‐9 (green), (**C**) CD31 (red) and (**D**) Cleaved caspase‐3 (green) level in laser‐injured area. Immunofluorescence images were captured by spinning disc confocal microscope. The activated caspase‐3‐positive cells were indicated by red arrows. Original magnification 20×, Bar = 50 μm. The laser lesions are marked by star. (**E**) Iba1^+^ cells detection at laser‐injured retina. Iba1 immunohistochemical staining images were captured by Zeiss microscope. Original magnification 20× Bar = 50 μm. Three images from at least two eyes representative of each group are included in this study.

VEGF‐A and C5b‐9, also known as membrane attack complex (MAC), have been reported to be increased in different CNV models 23, 37. In our study, analysis of immunofluorescence staining showed that AAV‐VID and AAV‐ACVP1 were able to reduce VEGF‐A level (Fig. 7A and Fig. S3d) in the local laser‐injured retina which was confirmed by immunoblot analysis (Fig. S3a–c) and formation of C5b‐9 in the choroid could be inhibited after intravitreal administration of AAV‐CID and AAV‐ACVP1 (Fig. 7B and Fig. S3e). CD31 staining of endothelial cells indicated that AAV‐VID and AAV‐ACVP1 could significantly suppress angiogenesis by blocking VEGF in CNV (Fig. 7C and Fig. S3f). Iba‐1 staining of microglia and macrophages showed that possible vascular inflammation occurred in the retina of untreated and control vector‐treated group. However, Iba+ infiltrated cells were significantly reduced in all groups treated with vectors expressing inhibitors. AAV‐ACVP1 provided a much better protection than the other two vectors (Fig. 7E and Fig. S3h). Upon investigating the apoptosis in mouse retina, scattered caspase‐3 positive cells were observed at the site of local laser injury (Fig. 7D and Fig. S3g). The retinas that received AAV‐VID or AAV‐CID had less cellular apoptosis compared to the untreated or control vector‐treated eyes. There was no visible apoptosis in retinas treated with AAV‐ACVP1, which suggests that sufficient protection could be conferred in the laser‐induced CNV model if both VEGF pathway and complement cascade were blocked.

### Effects of long‐term overexpression of CID, VID and ACVP1 from AAV vectors in normal mouse eyes

**Figure 8:**
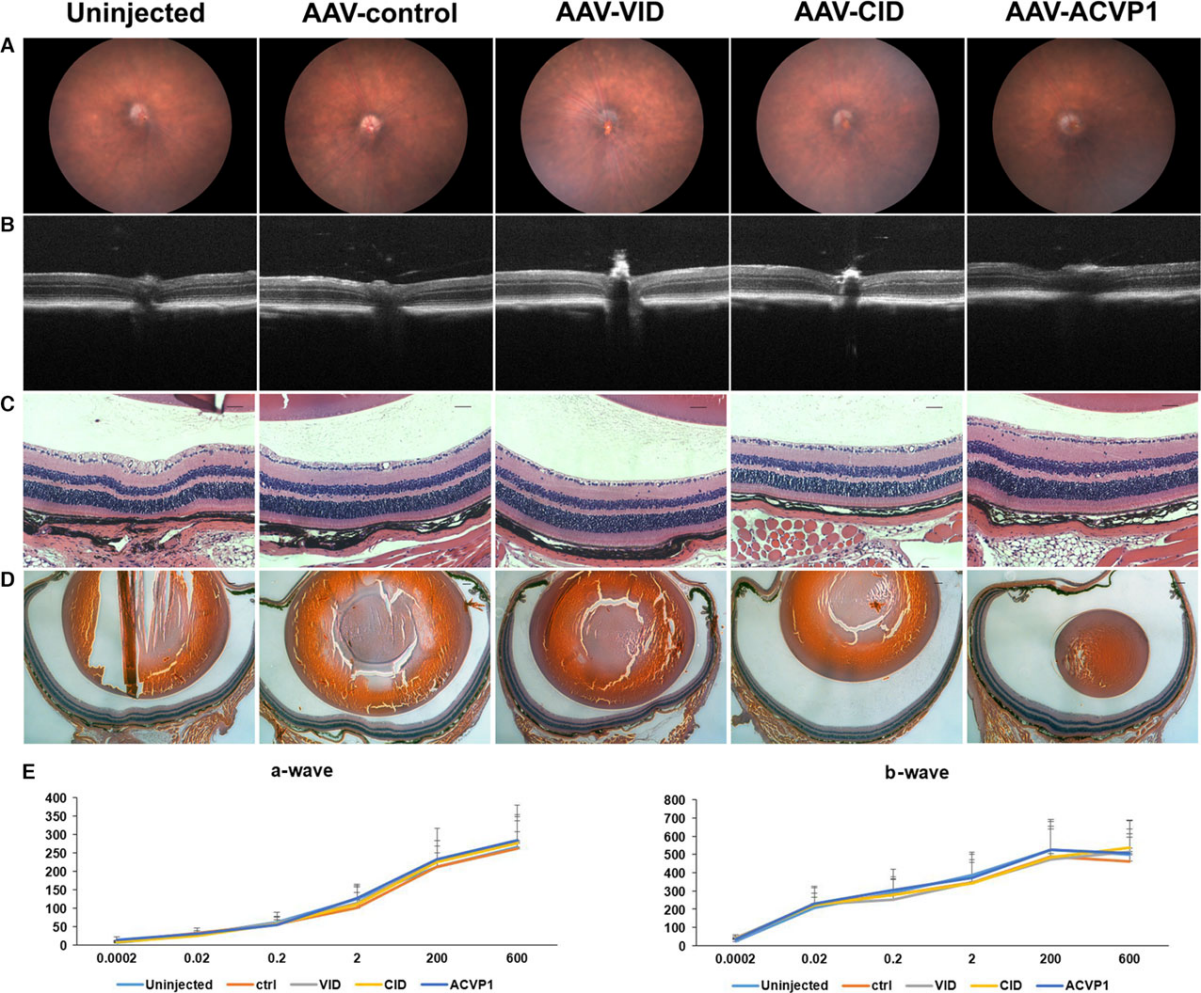
Evaluation of the effects of intravitreal administered VEGF inhibitor domain (VID), complement inhibitor domain (CID) and ACVP1 AAV vectors on retinal structure and visual function in wild‐type mice. (**A**) Representative fundoscopy images of C57BL/6J mice 4 months after intravitreal AAV vector administration from different groups. (**B**) Representative OCT images of C57BL/6J mice 4 months after intravitreal administration. (**C**) Representative H&E staining images of mouse retina. Original magnification 20× Bar = 50 μm. (**D**) Representative histological images of mouse eyes. Original magnification 2.5× Bar = 200 μm. (**E**) Full‐field scotopic ERG recordings of a and b waves. Values on *y*‐axis represent ERG response of a‐wave and b‐wave amplitude (uV). Values on *x*‐axis represent flash luminance (cd s/m^2^). Results are given as mean ± SD; (*n* = 5 per group).

As VEGF may have neuroprotective role, some studies showed that long‐term inhibition may be detrimental to retinal visual function 38. In our study, we investigated the potential effects of this AAV vector treatment on retinal structure and visual function in normal C57BL6J mice. Fundoscopy (Fig. 8A) and OCT (Fig. 8B) was analysed four months after AAV treatment administrated at the same doses used in previous studies. No significant difference was observed between AAV‐injected groups and uninjected group. Similar results were obtained by electroretinogram (ERG) evaluation (Fig. 8E). Histologically, no retinal degeneration, haemorrhage or detachment were observed in any of the vector‐treated group (Fig. 8C,D).

## Discussion

Both VEGF pathway and complement system play important roles in various ocular diseases. Several biological therapeutics targeting VEGF or complement system separately have been studied in the clinical trials with encouraging results. So far, a combination of anti‐VEGF and anti‐complement therapeutics has not been tried, but it may have a great treatment potential. In this study, we developed a novel molecule that blocks both VEGF and complement activation cascade by binding to both VEGF‐A and complement component C3b/C4b and tested its anti‐inflammatory and anti‐angiogenic efficacy *in vivo*. Our results suggest that this dual inhibitor molecule ameliorated ocular inflammation in animal models of uveitis and reduce laser‐induced choroidal neovascularization in mice.

Endotoxin induced uveitis has been widely used as an animal model to evaluate both anterior and posterior uveitis. Our results showed that inhibition of complement system suppressed the endotoxin‐induced ocular inflammation which is consistent with previous study 39. It has been reported that leucocytes are rapidly attracted to inflamed ocular tissues such as the iris 40 and vitreous cavity 41, and neutrophils, monocytes and macrophages are the major leucocyte constituents in EIU model. Immunostaining of these antigens indicated that the recruitment of different types of infiltrating inflammatory cells was inhibited by blocking the complement cascade. The vector expressing VEGF inhibitor alone did not seem to mitigate the ocular inflammation compared to the dual inhibitor or the vector expressing complement inhibitor, suggesting that VEGF may not play major role in pathogenesis of this particular model.

Experimental autoimmune uveitis is also used to evaluate all the vectors in this study. Unlike EIU, the EAU is initiated by an antigen‐specific T cell‐mediated intraocular inflammation and mostly affects the posterior segment of the eye. The process of the inflammation is much more chronic and persistent comparing to uveitis induced by lipopolysaccharide. The histopathological evaluation showed that AAV‐CID and AAV‐ACVP1 vector treatment provided protection from autoimmune uveitis, consistent with previous studies 42, suggesting that complement cascade is involved in ocular inflammation of both EIU and EAU. Interestingly, non‐invasive evaluation by fundoscopy and OCT imaging indicated that AAV‐VID also showed some beneficial effects with mild retinal folds, vasculitis and reduced retinal thickness caused by retinal folds and oedema in EAU model, which is different from results seen in EIU eyes. This result suggests that VEGF may also be involved in pathogenesis of EAU, or it may participate in the process of tissue repair and remodelling. Thus, dual inhibition of both complement cascade and VEGF could be considered as an alternative option in the chronic uveitis.

The AAV vectors were also investigated in the laser‐induced CNV model, which has been most established and extensively employed in the studies of wet age‐related macular degeneration. Laser‐induced CNV assay has been reported to be capable of recapitulating the complex biological process involved in the exudative form of AMD disease 37. Numerous components of angiogenesis 43 and inflammation 44 have been studied in this model, and the results established the early proof for the pharmacotherapy of CNV 17, 35. Numerous studies have shown that combined therapy has great potential in this model 45, 46. For viral vector‐mediated gene delivery, combined therapy would be more efficient with less immune response to viral vectors. Our results validated that inhibition of either VEGF or complement pathway mediated by AAV gene therapy ameliorated the CNV development. The VEGF level and retinal angiogenesis could not be significantly inhibited by our complement inhibitor based on our results, although our complement inhibitor could significant reduce the vascular leaking and choroid neovascularization. We speculate that unlike VEGF, which plays a core role in angiogenesis and neovascularization, the complement factors, especially anaphylatoxins regulate neovascularization moderately depending on various other chemokines. As complement components are involved in the pathogenesis of dry AMD, it is expected that our dual inhibitor could achieve beneficial effect on both dry and wet AMD. Further, for the first time VEGF and complement pathways were simultaneously blocked in CNV animal model, and beneficial effects of the dual inhibitor were much more significant than either inhibitor used alone, which indicates that both VEGF and complement pathway both contribute to the pathological progression of CNV.

Increasing evidence indicates that both angiogenesis and inflammation cooperate with each other 47, 48 and contribute to the aetiology of disease. Thus, numerous therapeutic agents targeting the components of angiogenesis or inflammation have been developed and examined in the experimental or clinical trials 49, 50. Our study for the first time combined anti‐angiogenesis and anti‐inflammation treatment by AAV delivered gene therapy and examined this treatment in the murine model. According to the therapeutic effect in the acute uveitis EIU model, the dual inhibitor (ACVP1) is not significantly superior to the CID. However, dual inhibitor provided more improvement compared to either VEGF inhibitor or complement inhibitor when applied in the autoimmune uveitis model and CNV model. Based on the results, we speculated that VEGF and complement cascade play different roles in the process of inflammation.

Gene therapy has been used in different diseases. The next generation of treatment for ocular diseases must demonstrate longer‐term, wider efficacy and reduce the need for frequent administrations 51. Several reports have shown beneficial effects of gene therapy with VEGF or complement inhibitors in different animal models 52. Eye is considered as an attractive organ for gene therapy due to its small compartment size, easy to visualize and examine and relatively immune‐privileged. AAV vectors have been approved to be an ideal tool in gene therapy because of their attractive features including low toxicity, lack of pathogenesis, efficient infection in different cells and tissues and long‐term gene expression. Whilst still in its experimental and early clinical trial stages, gene therapy delivered by AAV appears to have many advantages comparing to current intravitreal administration modalities. In this study, the chicken β‐actin promoter and AAV‐2 (sext Y‐F) variant were used as this capsid‐modified AAV2 serotype transduced retinal cells more efficiently than wild‐type AAV2 31, 32, so that lower viral titre could be used. Nevertheless, other capsid‐modified AAV serotypes and cell‐specific promoters could be further explored in future studies. Our results in this study demonstrated that inhibitor genes delivered by recombinant AAV could be expressed and secreted constantly with therapeutic efficacy in three different ocular inflammation and neovascular disease models. Furthermore, intravitreal administration of AAV vectors expressing these inhibitor genes did not show any toxic effects based on histological and ERG analysis (Fig. 8), and thus may be safe for clinical application. However, future studies are required to determine minimal effective doses and safety for long‐term application in large animals.

In conclusion, this study provides further proof that angiogenesis and inflammation are involved in aetiology of uveitis and CNV. The bispecific molecule blocking both VEGF and complement component delivered by AAV vector may offer sustained therapeutic effect for a wide range of ocular inflammatory and neovascular diseases.

## Conflict of interest

The authors confirm that there are no conflicts of interest.

## Supporting information


**Figure S1** Single intravitreal injection of capsid modified mutant AAV2 vector (1μl, 10^9^ vg/eye) resulted in efficient transduction of retinal cells.
**Figure S2** Funduscopic evaluation immediately after laser injuries and quantitative evaluation of CNV by fluorescein angiography and immunostaining.
**Figure S3** Evaluation of the VEGF level in retina of laser induced CNV mice by Western blotting.

## References

[1] Campochiaro PA . Ocular neovascularization. J Mol Med (Berl). 2013; 91: 311–21. 23329331 10.1007/s00109-013-0993-5 PMC3584193

[2] Lee K , Bajwa A , Freitas‐Neto CA , *et al* A comprehensive review and update on the non‐biologic treatment of adult noninfectious uveitis: part I. Expert Opin Pharmacother. 2014; 15: 2141–54. 25226529 10.1517/14656566.2014.948417

[3] Brady CJ , Villanti AC , Law HA , *et al* Corticosteroid implants for chronic non‐infectious uveitis. Cochrane Database Syst Rev. 2016; (2): CD010469. 26866343 10.1002/14651858.CD010469.pub2 PMC5038923

[4] Leung TG , Thorne JE . Emerging drugs for the treatment of uveitis. Expert Opin Emerg Drugs. 2013; 18: 513–21. 24274613 10.1517/14728214.2013.861417

[5] Dunn JP . Uveitis. Prim Care. 2015; 42: 305–23. 26319340 10.1016/j.pop.2015.05.003

[6] Uchiyama E , Papaliodis GN , Lobo AM , *et al* Side‐effects of anti‐inflammatory therapy in uveitis. Semin Ophthalmol. 2014; 29: 456–67. 25325874 10.3109/08820538.2014.959203

[7] Bansal S , Barathi VA , Iwata D , *et al* Experimental autoimmune uveitis and other animal models of uveitis: an update. Indian J Ophthalmol. 2015; 63: 211–8. 25971165 10.4103/0301-4738.156914 PMC4448233

[8] Weiss K , Steinbrugger I , Weger M , *et al* Intravitreal VEGF levels in uveitis patients and treatment of uveitic macular oedema with intravitreal bevacizumab. Eye (Lond). 2009; 23: 1812–8. 19169227 10.1038/eye.2008.388

[9] Perez VL , Caspi RR . Immune mechanisms in inflammatory and degenerative eye disease. Trends Immunol. 2015; 36: 354–63. 25981967 10.1016/j.it.2015.04.003 PMC4563859

[10] Camelo S . Potential sources and roles of adaptive immunity in age‐related macular degeneration: shall we rename AMD into autoimmune macular disease? Autoimmune Dis. 2014; 2014: 532487. 24876950 10.1155/2014/532487 PMC4022009

[11] Chen M , Xu H . Parainflammation, chronic inflammation, and age‐related macular degeneration. J Leukoc Biol. 2015; 98: 713–25. 26292978 10.1189/jlb.3RI0615-239R PMC4733662

[12] Wang Y , Wang VM , Chan CC . The role of anti‐inflammatory agents in age‐related macular degeneration (AMD) treatment. Eye (Lond). 2011; 25: 127–39. 21183941 10.1038/eye.2010.196 PMC3044916

[13] van Lookeren Campagne M , LeCouter J , Yaspan BL , *et al* Mechanisms of age‐related macular degeneration and therapeutic opportunities. J Pathol. 2014; 232: 151–64. 24105633 10.1002/path.4266

[14] Siemerink MJ , Augustin AJ , Schlingemann RO . Mechanisms of ocular angiogenesis and its molecular mediators. Dev Ophthalmol. 2010; 46: 4–20. 20703029 10.1159/000320006

[15] Kinnunen K , Yla‐Herttuala S . Vascular endothelial growth factors in retinal and choroidal neovascular diseases. Ann Med. 2012; 44: 1–17. 21284527 10.3109/07853890.2010.532150

[16] Stuart A , Ford JA , Duckworth S , *et al* Anti‐VEGF therapies in the treatment of choroidal neovascularisation secondary to non‐age‐related macular degeneration: a systematic review. BMJ Open. 2015; 5: e007746. 10.1136/bmjopen-2015-007746 PMC4420986 25941188

[17] Amadio M , Govoni S , Pascale A . Targeting VEGF in eye neovascularization: What's new?: a comprehensive review on current therapies and oligonucleotide‐based interventions under development. Pharmacol Res. 2016; 103: 253–69. 26678602 10.1016/j.phrs.2015.11.027

[18] Gulati N , Forooghian F , Lieberman R , *et al* Vascular endothelial growth factor inhibition in uveitis: a systematic review. Br J Ophthalmol. 2011; 95: 162–5. 20494915 10.1136/bjo.2009.177279

[19] Read RW , Szalai AJ , Vogt SD , *et al* Genetic deficiency of C3 as well as CNS‐targeted expression of the complement inhibitor sCrry ameliorates experimental autoimmune uveoretinitis. Exp Eye Res. 2006; 82: 389–94. 16143328 10.1016/j.exer.2005.07.011

[20] Jha P , Sohn JH , Xu Q , *et al* The complement system plays a critical role in the development of experimental autoimmune anterior uveitis. Invest Ophthalmol Vis Sci. 2006; 47: 1030–8. 16505038 10.1167/iovs.05-1062 PMC1975680

[21] Nozaki M , Raisler BJ , Sakurai E , *et al* Drusen complement components C3a and C5a promote choroidal neovascularization. Proc Natl Acad Sci U S A. 2006; 103: 2328–33. 16452172 10.1073/pnas.0408835103 PMC1413680

[22] Lipo E , Cashman SM , Kumar‐Singh R . Aurintricarboxylic acid inhibits complement activation, membrane attack complex, and choroidal neovascularization in a model of macular degeneration. Invest Ophthalmol Vis Sci. 2013; 54: 7107–14. 24106121 10.1167/iovs.13-12923 PMC3813320

[23] Bora PS , Sohn JH , Cruz JM , *et al* Role of complement and complement membrane attack complex in laser‐induced choroidal neovascularization. J Immunol. 2005; 174: 491–7. 15611275 10.4049/jimmunol.174.1.491

[24] Heier JS , Brown DM , Chong V , *et al* Intravitreal aflibercept (VEGF trap‐eye) in wet age‐related macular degeneration. Ophthalmology. 2012; 119: 2537–48. 23084240 10.1016/j.ophtha.2012.09.006

[25] Wykoff CC , Hariprasad SM . Comparing aflibercept, bevacizumab, and ranibizumab for DME: analysis of DRCR Protocol T. Ophthalmic Surg Lasers Imaging Retina. 2015; 46: 302–5. 25835307 10.3928/23258160-20150304-01

[26] Shil PK , Kwon KC , Zhu P , *et al* Oral delivery of ACE2/Ang‐(1‐7) bioencapsulated in plant cells protects against experimental uveitis and autoimmune uveoretinitis. Mol Ther. 2014; 22: 2069–82. 25228068 10.1038/mt.2014.179 PMC4429699

[27] Agarwal RK , Silver PB , Caspi RR . Rodent models of experimental autoimmune uveitis. Methods Mol Biol. 2012; 900: 443–69. 22933083 10.1007/978-1-60761-720-4_22 PMC3810964

[28] Lambert V , Lecomte J , Hansen S , *et al* Laser‐induced choroidal neovascularization model to study age‐related macular degeneration in mice. Nat Protoc. 2013; 8: 2197–211. 24136346 10.1038/nprot.2013.135

[29] Li Q , Dinculescu A , Shan Z , *et al* Downregulation of p22phox in retinal pigment epithelial cells inhibits choroidal neovascularization in mice. Mol Ther. 2008; 16: 1688–94. 18665154 10.1038/mt.2008.164 PMC4851833

[30] Chen J , Qian H , Horai R , *et al* Use of optical coherence tomography and electroretinography to evaluate retinal pathology in a mouse model of autoimmune uveitis. PLoS ONE. 2013; 8: e63904. 23691112 10.1371/journal.pone.0063904 PMC3653843

[31] Petrs‐Silva H , Dinculescu A , Li Q , *et al* Novel properties of tyrosine‐mutant AAV2 vectors in the mouse retina. Mol Ther. 2011; 19: 293–301. 21045809 10.1038/mt.2010.234 PMC3034844

[32] Petrs‐Silva H , Dinculescu A , Li Q , *et al* High‐efficiency transduction of the mouse retina by tyrosine‐mutant AAV serotype vectors. Mol Ther. 2009; 17: 463–71. 19066593 10.1038/mt.2008.269 PMC2835095

[33] Rosenbaum JT , McDevitt HO , Guss RB , *et al* Endotoxin‐induced uveitis in rats as a model for human disease. Nature. 1980; 286: 611–3. 7402339 10.1038/286611a0

[34] Copland DA , Wertheim MS , Armitage WJ , *et al* The clinical time‐course of experimental autoimmune uveoretinitis using topical endoscopic fundal imaging with histologic and cellular infiltrate correlation. Invest Ophthalmol Vis Sci. 2008; 49: 5458–65. 18757507 10.1167/iovs.08-2348

[35] Yehoshua Z , de Amorim Garcia Filho CA , Nunes RP , *et al* Systemic complement inhibition with eculizumab for geographic atrophy in age‐related macular degeneration: the COMPLETE study. Ophthalmology. 2014; 121: 693–701. 24289920 10.1016/j.ophtha.2013.09.044 PMC4015213

[36] Munk MR , Ruckert R , Zinkernagel M , *et al* The role of anti‐VEGF agents in myopic choroidal neovascularization: current standards and future outlook. Expert Opin Biol Ther. 2016; 16: 477–87. 26666589 10.1517/14712598.2016.1132696

[37] Huang H , Parlier R , Shen JK , *et al* VEGF receptor blockade markedly reduces retinal microglia/macrophage infiltration into laser‐induced CNV. PLoS ONE. 2013; 8: e71808. 23977149 10.1371/journal.pone.0071808 PMC3748119

[38] Scott AW , Bressler SB . Long‐term follow‐up of vascular endothelial growth factor inhibitor therapy for neovascular age‐related macular degeneration. Curr Opin Ophthalmol. 2013; 24: 190–6. 23492430 10.1097/ICU.0b013e32835fefee

[39] Rosenbaum JT , Wong K , Perez HD , *et al* Characterization of endotoxin‐induced C5‐derived chemotactic activity in aqueous humor. Invest Ophthalmol Vis Sci. 1984; 25: 1184–91. 6384121

[40] Planck SR , Becker MD , Crespo S , *et al* Characterizing extravascular neutrophil migration *in vivo* in the iris. Inflammation. 2008; 31: 105–11. 18196451 10.1007/s10753-007-9055-x

[41] Noda K , Miyahara S , Nakazawa T , *et al* Inhibition of vascular adhesion protein‐1 suppresses endotoxin‐induced uveitis. FASEB J. 2008; 22: 1094–103. 18032635 10.1096/fj.07-9377com

[42] Chen M , Muckersie E , Luo C , *et al* Inhibition of the alternative pathway of complement activation reduces inflammation in experimental autoimmune uveoretinitis. Eur J Immunol. 2010; 40: 2870–81. 20806290 10.1002/eji.201040323

[43] Prea SM , Chan EC , Dusting GJ , *et al* Gene therapy with endogenous inhibitors of angiogenesis for neovascular age‐related macular degeneration: beyond anti‐VEGF therapy. J Ophthalmol. 2015; 2015: 201726. 25821585 10.1155/2015/201726 PMC4363820

[44] Oshima Y , Oshima S , Nambu H , *et al* Increased expression of VEGF in retinal pigmented epithelial cells is not sufficient to cause choroidal neovascularization. J Cell Physiol. 2004; 201: 393–400. 15389527 10.1002/jcp.20110

[45] Robbie SJ , Lundh von Leithner P , Ju M , *et al* Assessing a novel depot delivery strategy for noninvasive administration of VEGF/PDGF RTK inhibitors for ocular neovascular disease. Invest Ophthalmol Vis Sci. 2013; 54: 1490–500. 23385800 10.1167/iovs.12-10169

[46] Askou AL , Aagaard L , Kostic C , *et al* Multigenic lentiviral vectors for combined and tissue‐specific expression of miRNA‐ and protein‐based antiangiogenic factors. Mol Ther Methods Clin Dev. 2015; 2: 14064. 26052532 10.1038/mtm.2014.64 PMC4449022

[47] Kim YW , West XZ , Byzova TV . Inflammation and oxidative stress in angiogenesis and vascular disease. J Mol Med (Berl). 2013; 91: 323–8. 23430240 10.1007/s00109-013-1007-3 PMC3656485

[48] Liu J , Jha P , Lyzogubov VV , *et al* Relationship between complement membrane attack complex, chemokine (C‐C motif) ligand 2 (CCL2) and vascular endothelial growth factor in mouse model of laser‐induced choroidal neovascularization. J Biol Chem. 2011; 286: 20991–1001. 21515678 10.1074/jbc.M111.226266 PMC3121483

[49] Stewart MW . Aflibercept (VEGF Trap‐eye): the newest anti‐VEGF drug. Br J Ophthalmol. 2012; 96: 1157–8. 22446028 10.1136/bjophthalmol-2011-300654 PMC3432488

[50] Yang CS , Hung KC , Huang YM , *et al* Intravitreal bevacizumab (Avastin) and panretinal photocoagulation in the treatment of high‐risk proliferative diabetic retinopathy. J Ocul Pharmacol Ther. 2013; 29: 550–5. 23495932 10.1089/jop.2012.0202 PMC3708621

[51] Boye SE , Boye SL , Lewin AS , *et al* A comprehensive review of retinal gene therapy. Mol Ther. 2013; 21: 509–19. 23358189 10.1038/mt.2012.280 PMC3642288

[52] Lipinski DM , Thake M , MacLaren RE . Clinical applications of retinal gene therapy. Prog Retin Eye Res. 2013; 32: 22–47. 22995954 10.1016/j.preteyeres.2012.09.001

