# Supplementary material for: A novel bispecific molecule delivered by recombinant AAV2 suppresses ocular inflammation and choroidal neovascularization
Source: J Cell Mol Med. 2017 Mar 22;21(8):1555–71. doi: 10.1111/jcmm.13086 (PMC5543459; doi:10.1111/jcmm.13086)
Supplement: Supplementary file 1 — Figure S1 Single intravitreal injection of capsid modified mutant AAV2 vector (1μl, 109 vg/eye) resulted in efficient transduction of retinal cells. Figure S2 Funduscopic evaluation immediately after laser injuries and quantitative evaluation of CNV by fluorescein angiography and immunostaining. Figure S3 Evaluation of the VEGF level in retina of laser induced CNV mice by Western blotting. [file JCMM-21-1555-s001.doc]

**A novel bispecific molecule delivered by recombinant AAV2 suppresses ocular inflammation and choroidal neovascularization**

Yiming Li^1, 2^, Ping Zhu^2^, Amrisha Verma^2^, Tuhina Prasad^2^, Hongxin Deng^1^, Dechao Yu^1*^ and Qiuhong Li^2*^

^1^State Key Laboratory of Biotherapy and Cancer center, West China Hospital, Sichuan University, and Collaborative Innovation Center for Biotherapy

^2^Department of Ophthalmology and Powell Gene Therapy Center, University of Florida College of Medicine, Gainesville, Florida, USA

**Corresponding Author:**

Qiuhong Li, Ph.D.

Department of Ophthalmology

University of Florida,

Gainesville, Florida 32610-0284, USA

E-mail: [qli@ufl.edu](mailto:qli@ufl.edu)

Telephone: 352-392-0747

Fax: 352-392-0573

Michael Yu, Ph.D.

State Key Laboratory of Biotherapy and Cancer center

Sichuan University

Chengdu, Sichuan 610041, China, People’s Republic of China

E-mail: michael.yu@innoventbio.com

**Supplementary material**

**Supplemental Figure 1**. Single intravitreal injection of capsid modified mutant AAV2 vector (1μl, 10^9^ vg/eye) resulted in efficient transduction of retinal cells. (a) Retinal whole mount, (b) cross section of a vector injected mouse eye at low magnification and (c) cross section of a vector injected mouse eye at low magnification.

**Supplemental Figure 2.** Funduscopic evaluation immediately after laser injuries and quantitative evaluation of CNV by fluorescein angiography and immunostaining.

(a) Representative fundus images in laser-induced CNV mice taken immediately after laser injuries. Different groups of mice randomly received laser treatment three weeks after AAV intravitreal administration. (b) Retinal vascular leakage was measured by fluorescence angiography. Values on y-axis represent leaking area measured by fluorescence images. (c) Lesion area induced by laser was relatively measured by fundoscopy. (d) Lesion area was measured by Zeiss AxioVision Software measurement tool from H&E images. Values on y-axis represent laser area. Results are given as mean + SD; (n = 5 per group); * means P < 0.05 (versus un-injected or AAV-control group) # means P<0.05(versus AAV-CID or AAV-VID group).

**Supplemental Figure 3.** Evaluation of the VEGF level in retina of laser induced CNV mice by Western blotting.

Retinas from different groups (Lane 1 Uninjected, Lane 2 AAV-control, Lane 3 AAV-CID, Lane 4 AAV-VID and Lane 5 AAV-ACVP1) were separated from eye cup once mice were sacrificed. Protein lysates were harvested and analyzed by Western blot probed with an antibody specific to VEGF (a) and β-actin (b). (c) Quantification of VEGF expression on retina based on Western blot. Results are given as mean + SD; * means P<0.05 (versus uninjected group). Values on y-axis represent relatively VEGF expression normalized to β-actin. (d) Quantification of VEGF expression of laser area based on immunostaining. (e) Quantification of C5b-9 accumulation in choroid. (f) Quantification of CD31+ endothelial cells from immunostaining sections. (g) Quantification of caspase-3 positive cells based on immunostaining. (h) Quantification of lba1^+^ microglia cells from immunostaining. Results are given as mean + SD; (n = 3 per group); * means P < 0.001 (versus uninjected group) # means P<0.01 (versus AAV-CID or AAV-VID group). Values on y-axis represent number of positive cells per section.

**Supplementary methods**

**VEGF binding assay**

Affinity of the dual inhibitor (ACVP1) to VEGF was determined by indirect enzyme-linked immune sorbent assay. ACVP1 (0-10nM) were incubated with immobilized VEGF165 (30nM). After washing, the bound proteins were detected by blocking with horseradish peroxidase-conjugated goat anti-human IgG Fc. After color development, optical density at 450 nm (OD450) was determined with an ELISA plate reader.

**Complement hemolysis assay**


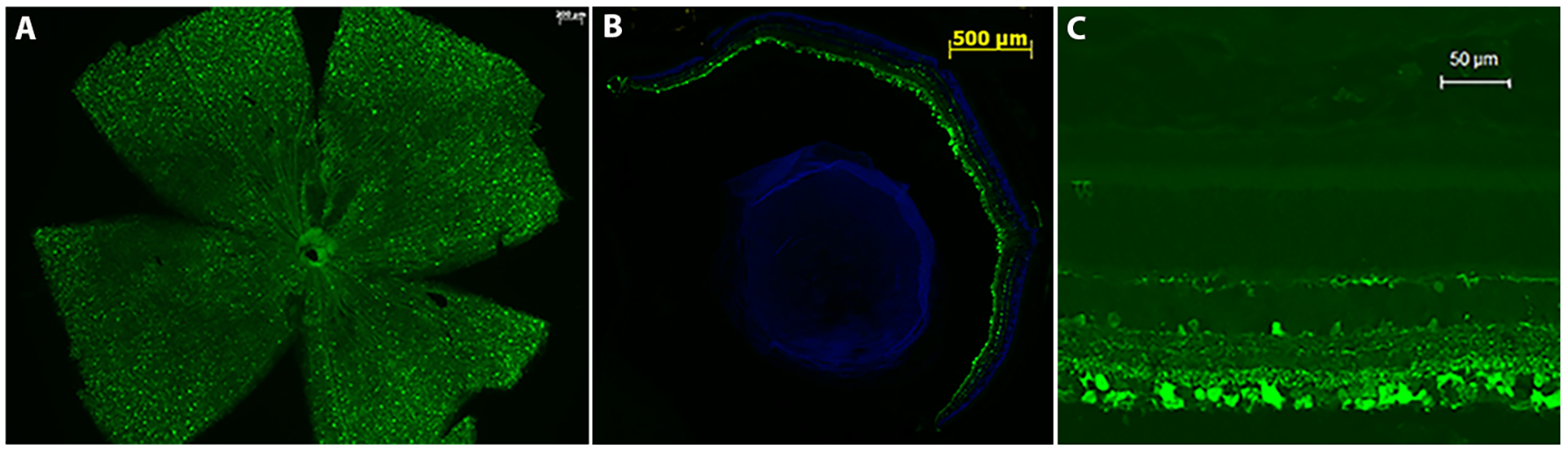
Inhibition efficiency of ACVP1 to complement cascade was determined by classical pathway hemolysis assay. ACVP1 (0-500nM) were incubated with diluted normal human serum. Next, mixture were added into 1 x 10^6^ antibody-sensitized sheep erythrocytes. 1hr later, hemolysis were assayed by absorption at OD 405 nm.

# Supplement Figure. 1

**Supplemental Figure. 2**


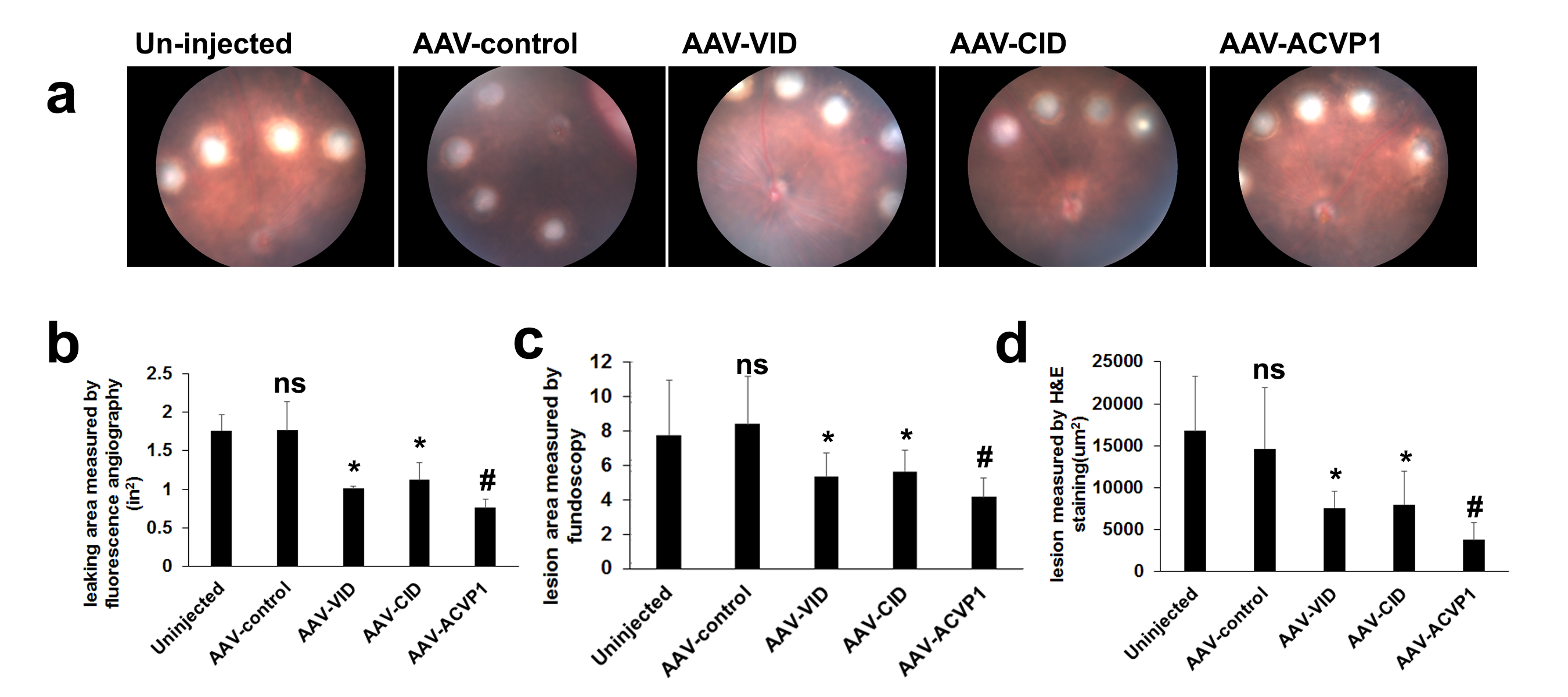


**Supplemental Figure. 3**

**
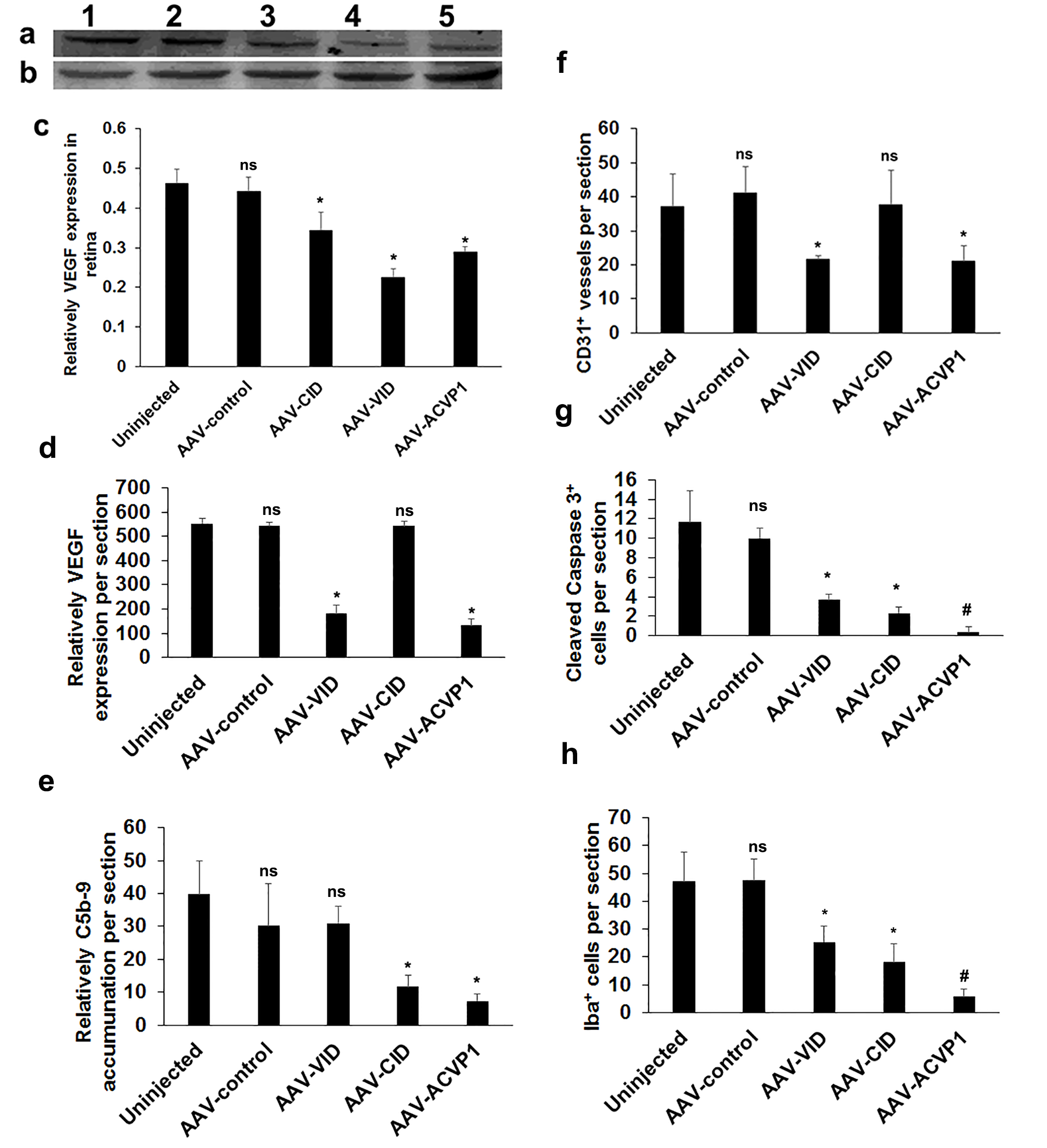
**
